# Frail, Fluid, and Falsely Accused: Myxedema Ascites in an Older Adult

**DOI:** 10.7759/cureus.115389

**Published:** 2026-08-29

**Authors:** Arun Sankar, Abinash Jha, Surjitkumar Haobam, Neethu Sunny, Monika Pathania

**Affiliations:** 1 Geriatric Medicine, All India Institute of Medical Sciences Rishikesh, Rishikesh, IND; 2 Internal Medicine, All India Institute of Medical Sciences Rishikesh, Rishikesh, IND

**Keywords:** frail elderly, geriatric patient, hypothyroidism, myxedema ascites, saag

## Abstract

Ascites is a rare presentation of hypothyroidism and can be easily mistaken for malignancy when it occurs alongside pleural effusion and radiological lymphadenopathy. We report the case of a 63-year-old man who presented with progressive generalized anasarca, abdominal distension, and worsening breathlessness. Diagnostic paracentesis revealed exudative ascites with a low serum-ascites albumin gradient (SAAG) and markedly elevated protein content, and thyroid function testing showed profound, previously undiagnosed primary hypothyroidism (thyroid-stimulating hormone (TSH) 221.2 µIU/mL). On a subsequent admission, contrast-enhanced CT additionally revealed bilateral pleural effusion with mediastinal lymphadenopathy and omental fat stranding, raising concern for an underlying malignant or granulomatous process. Repeat ascitic fluid analysis, cytology, tumor markers, and cartridge-based nucleic acid amplification test (CBNAAT) were unremarkable, and an omental biopsy was performed and was not suggestive of inflammatory pathology; in view of this reassuring result, together with the patient’s frailty and early clinical improvement, the higher-risk procedure, mediastinal lymph node biopsy, was deferred by multidisciplinary consensus in favor of a trial of thyroid hormone replacement. This strategy proved correct: with initiation and titration of levothyroxine, the patient’s ascites, pleural effusion, and edema resolved completely on serial follow-up, obviating the need for mediastinal lymph node sampling. This case illustrates that myxedema ascites can radiologically mimic malignancy and that thyroid function testing, together with judicious, risk-stratified tissue sampling and, where clinically appropriate, a closely monitored trial of thyroid hormone replacement, can help a frail older adult avoid an unnecessary high-risk biopsy.

## Introduction

Primary hypothyroidism is common and can present with protean manifestations ranging from cold intolerance and fatigue to, rarely, serosal fluid accumulation in the form of pericardial effusion, pleural effusion, and ascites [1,2]. Ascites attributable to hypothyroidism occurs in less than 4% of hypothyroid patients, and hypothyroidism itself accounts for only 1-5% of all cases of ascites [3,4]. Because ascites is seldom the presenting or dominant feature of hypothyroidism, the diagnosis is frequently delayed, and patients often undergo extensive, sometimes invasive, evaluation for more common causes such as cirrhosis, malignancy, tuberculosis, and cardiac failure before hypothyroidism is considered [5]. This delay carries particular consequences in older adults, in whom the diagnostic net widens further to include age-related malignancies and in whom frailty heightens the risks of invasive procedures. We describe a 63-year-old man with previously undiagnosed, severe hypothyroidism who presented across two admissions with anasarca, low-serum-ascites albumin gradient (SAAG) exudative ascites, and pleural effusion with radiological findings that raised concern for malignancy, illustrating both the diagnostic pitfalls and the near-complete reversibility of myxedema ascites with thyroid hormone replacement.

## Case presentation

A 63-year-old man, a chronic alcohol user and smoker with a three-year history of diabetes mellitus on treatment, presented with a two-week history of painless, symmetrical swelling of the face and bilateral lower limbs progressing to generalized body swelling, accompanied by gradually progressive, painless abdominal distension over 15 days and worsening breathlessness (modified Medical Research Council (mMRC) dyspnea grade II to IV) [6]. He denied chest pain, cough, orthopnea, or paroxysmal nocturnal dyspnea. He was independent in activities of daily living prior to this illness.

On examination at first admission, he was afebrile with a heart rate of 54/min (bradycardic), blood pressure 145/68 mmHg, and oxygen saturation 98% on room air. He had pallor with non-pitting pedal edema; icterus, cyanosis, clubbing, and lymphadenopathy were absent. Cardiovascular examination revealed normal heart sounds without a gallop. Respiratory examination showed bilateral fine inspiratory crepitations over the infrascapular regions. The abdomen was soft and distended with a positive fluid thrill and normal bowel sounds; no organomegaly or other stigmata of chronic liver disease were noted. He was assessed as mildly frail (Clinical Frailty Scale 5) [7] with probable sarcopenia (European Working Group on Sarcopenia in Older People 2 (EWGSOP2) criteria) [8] on comprehensive geriatric assessment. The clinical findings at presentation are shown in Figure 1.

**Figure 1:**
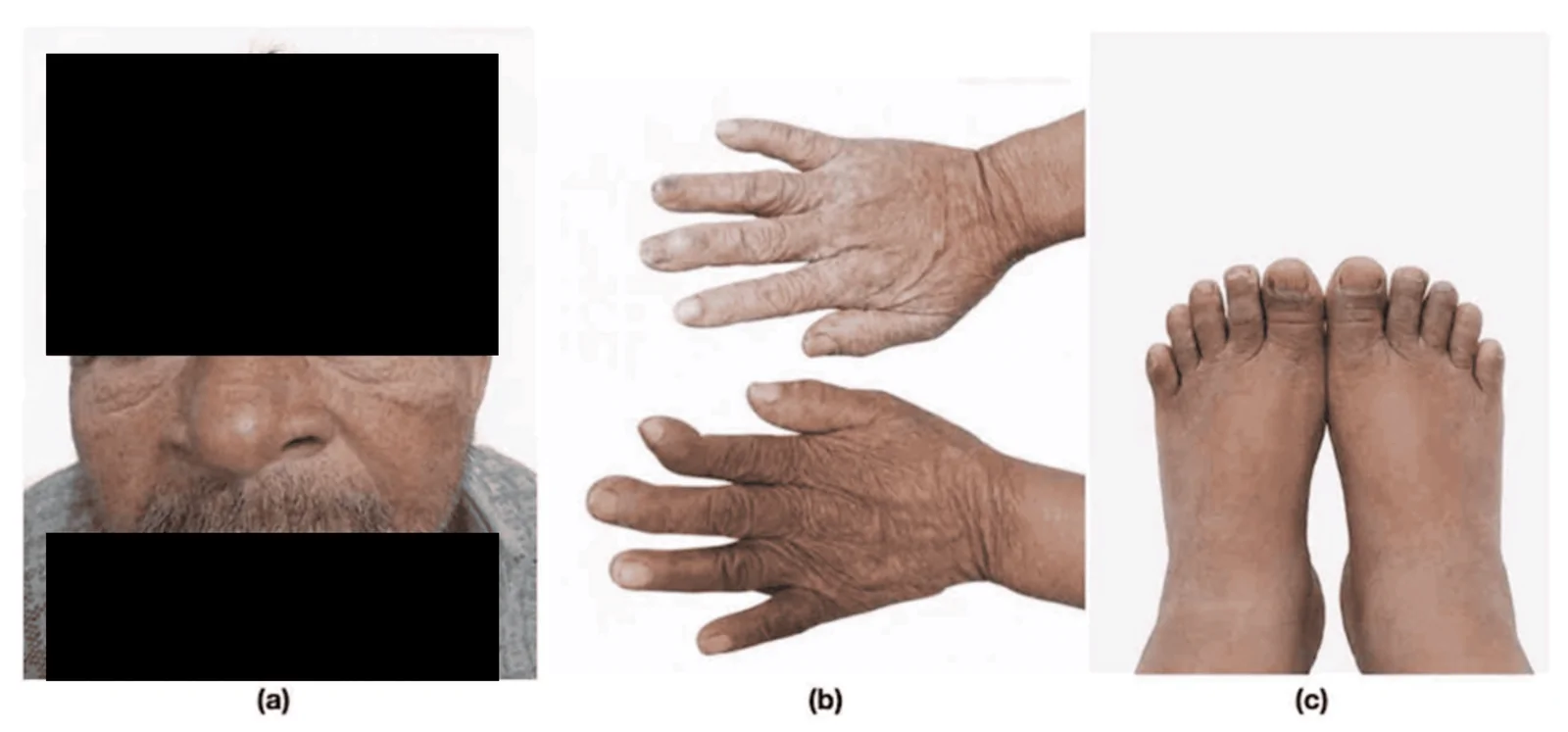
Clinical findings at initial presentation a: facial photograph showing periorbital puffiness, coarse facial features, and generalized non-pitting facial swelling characteristic of myxedema; b: dorsal aspect of both hands showing dry, coarse, thickened skin with non-pitting swelling of the fingers; c: both feet showing symmetrical non-pitting pedal edema. Written informed consent to include this image in an open-access article was obtained from the patient.

Initial investigations revealed normocytic normochromic anemia (hemoglobin 8.5 g/dL), renal parameters showing a disproportionate elevation of blood urea relative to creatinine, a pattern consistent with pre-renal azotemia, and elevated transaminases (aspartate aminotransferase (AST) 211 U/L, alanine aminotransferase (ALT) 102 U/L) with normal coagulation. Serum sodium was 137 mmol/L at admission but declined progressively to 119 mmol/L over the first three days of hospitalization, with urine osmolality of 455 mOsm/kg, prompting evaluation for a hyponatremic, dilutional state. While this pattern is a recognized biochemical accompaniment of severe hypothyroidism, the diuretic therapy administered during this period and the associated fluid shifts were also likely contributory, and the fall in sodium was therefore not attributed to hypothyroidism alone. An electrocardiogram showed normal sinus rhythm with low-voltage QRS complexes, and two-dimensional echocardiography demonstrated normal left ventricular systolic function with grade I diastolic dysfunction and no pericardial effusion or valvular abnormality, findings that, together with the absence of ischemic symptoms, made cardiac failure and significant structural cardiac dysfunction unlikely to be the primary cause of his anasarca [9]. The trend of key clinical and biochemical parameters across both admissions is summarized in Table 1.

**Table 1:** Trend of key clinical and biochemical parameters across both admissions TSH: thyroid-stimulating hormone; AST: aspartate aminotransferase; ALT: alanine aminotransferase

Parameter (normal range)	Admission 1 Day 1	Admission 1 Day 4	Admission 2 Day 1	Comment
Hemoglobin (12–15 g/dL)	8.5	9.4	9.8	Normocytic normochromic
TSH (0.35–5.5 µIU/mL)	221.2 (markedly elevated)	—	104	Consistent with severe primary hypothyroidism
AST / ALT (0–35 U/L)	211 / 102	118 / 31	49 / 17	Downtrending with thyroxine replacement
Serum albumin (3.5–5.2 g/dL)	3.5	3.8	3.5	—
Serum sodium (136–146 mmol/L)	137	119	130	Hyponatremia improved during admission 2; urine osmolality 455 mOsm/kg
Heart rate (bpm)	54	—	91	Bradycardia resolved after thyroxine initiation

Diagnostic and therapeutic paracentesis yielded exudative ascitic fluid with an elevated protein content of 3.7 g/dL, a low SAAG of 0.5 g/dL [10], and a lymphocyte-predominant cell count of 40/µL. Gram stain, cartridge-based nucleic acid amplification test (CBNAAT), and bacterial cultures of the ascitic fluid were negative, and cytology showed no malignant cells. Abdominal ultrasonography demonstrated altered liver echotexture with moderate ascites, but no focal hepatic lesion, and urine microscopy and culture did not support a renal or infective cause. Viral hepatitis serology was negative, coagulation parameters, including international normalized ratio (INR), were within normal limits, platelet count was normal, and portal venous Doppler was unremarkable, making chronic liver disease with portal hypertension an unlikely primary cause of the ascites. Given the low-SAAG picture, disseminated tuberculosis was also considered, and contrast-enhanced CT of the abdomen was planned to further evaluate this possibility. Thyroid function testing was markedly abnormal, with a thyroid-stimulating hormone (TSH) of 221.2 µIU/mL, confirming severe, previously undiagnosed primary hypothyroidism. During this hospitalization, he was also managed for onychomycosis with chronic hand eczema, for which a dermatology opinion was sought and topical therapy initiated. Levothyroxine was initiated at 100 mcg once daily, along with antihypertensives and supportive geriatric care. During the course of hospitalization, he showed symptomatic improvement and was discharged in a hemodynamically stable condition.

The patient re-presented ten days later with a urinary tract infection accompanied by abdominal discomfort. Contrast-enhanced CT at this admission revealed bilateral pleural effusion together with multiple heterogeneously enhancing mediastinal lymph nodes and omental fat stranding, prompting concern for an occult malignant or granulomatous disorder (Figure 2). On further work-up, repeat analysis of the ascitic fluid, including cytology, tumor markers, and CBNAAT, again showed no evidence of malignancy or tuberculosis (Table 2).

**Figure 2:**
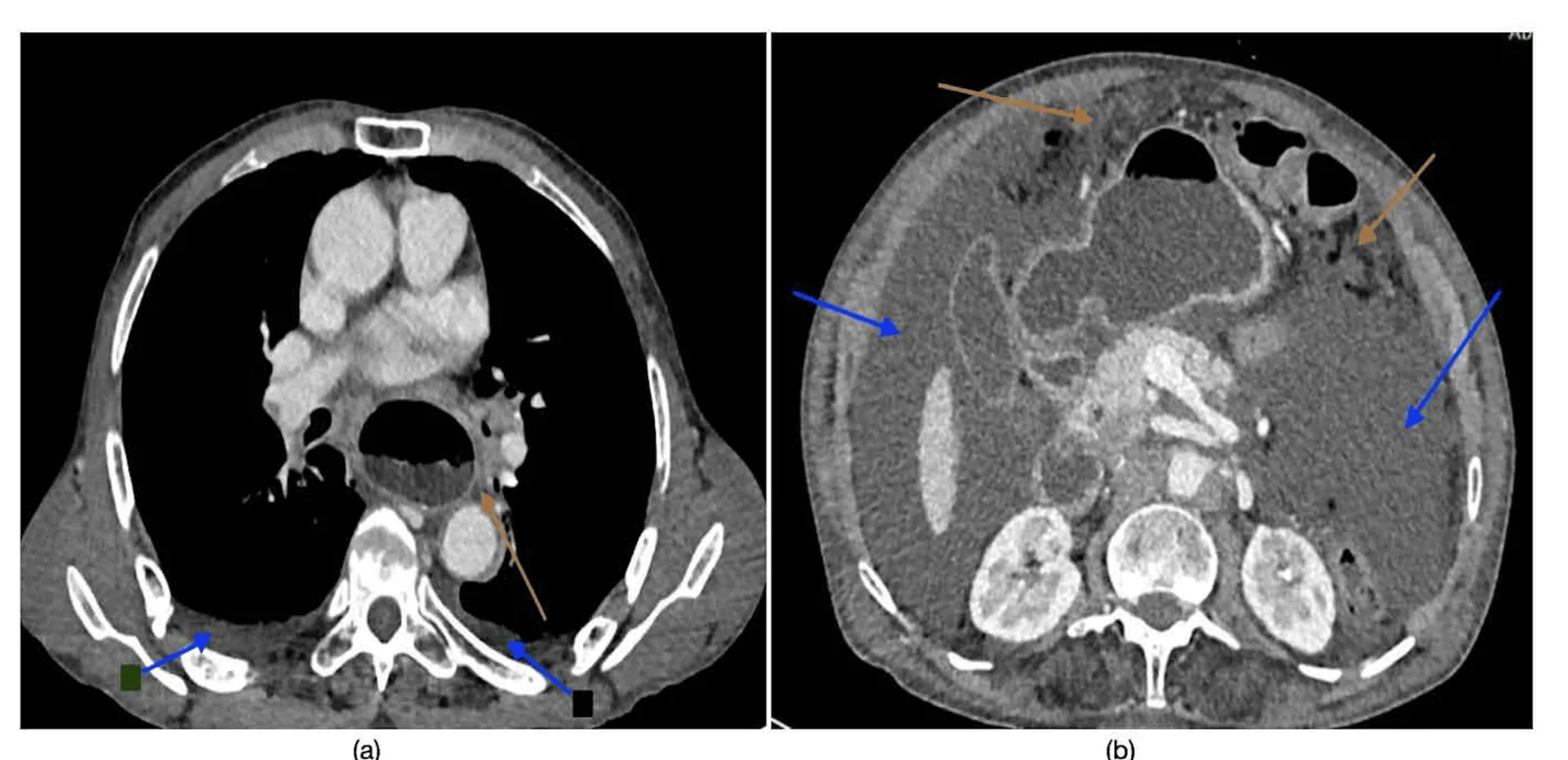
Contrast-enhanced CT at the second admission a: axial CT thorax showing bilateral pleural effusion (blue arrows) and heterogeneously enhancing subcarinal lymph nodes (green arrow); b: axial CT abdomen showing gross ascites (blue arrows) with omental nodularity and fat stranding (green arrows).

**Table 2:** Ascitic fluid characteristics compared with the literature *Literature comparison data adapted from Khalid et al. [11], reviewing 51 previously reported cases of myxedema ascites. SAAG: serum-ascites albumin gradient; WBC: white blood cell; CBNAAT: cartridge-based nucleic acid amplification test

Parameter	Literature (51 reported cases)*	Present case — Admission 1	Present case — Admission 2
Ascitic protein (g/dL)	Mean 3.9 (range 1.8-5.1)	3.7	3.4
SAAG (g/dL)	Mean 1.5 (range 0.8-2.3)	0.5	Not repeated
Ascitic WBC count (/µL)	Mean 60 (range 10-400)	40 (lymphocyte-predominant)	50 (lymphocyte-predominant)
Cytology / Gram stain / CBNAAT / culture	—	Negative for malignant cells and organisms; CBNAAT negative	Negative for malignant cells

Given the persistent omental stranding, an ultrasound-guided percutaneous omental biopsy was performed. The specimen consisted of multiple grey-white soft tissue cores measuring 0.1-0.4 cm. Microscopy showed fibrocollagenous tissue and adipose tissue focally lined by mesothelial cells, with a chronic lymphocytic infiltrate within the connective tissue septa between the fat lobules. No granuloma, necrosis, or cytological atypia was identified, and the appearances were reported as non-specific inflammatory change, providing further reassurance against a granulomatous or malignant process. In view of the patient’s frailty, early clinical improvement following thyroid hormone replacement, and this absence of supportive histological and biochemical evidence for malignancy or infection, the planned mediastinal lymph node biopsy, a comparatively higher-risk procedure in this frail patient, was deferred by multidisciplinary consensus. He was treated for the urinary tract infection with appropriate antibiotics. On this admission, his heart rate had normalized to 91/min, and pedal edema had resolved, reflecting an early response to thyroid hormone replacement; levothyroxine was up-titrated to 125 mcg once daily. He was discharged hemodynamically stable with plans for outpatient review of ascitic fluid cytology, tumor markers, CBNAAT, and repeat thyroid function.

On outpatient follow-up, coronary artery disease was reasonably excluded on the basis of the electrocardiogram, which showed no ischemic changes, and preserved left ventricular systolic function without regional wall motion abnormality. Levothyroxine was serially adjusted according to repeat thyroid function tests, and the patient’s ascites, pleural effusion, and residual edema resolved completely within two months, without further need for diuretics or repeat paracentesis (Figure 3). Over the following two months, levothyroxine was gradually tapered, and the patient was maintained on 75 mcg once daily, adjusted according to body weight and thyroid function, mirroring the pattern of rapid reversibility classically described in myxedema ascites once euthyroid status is restored.

**Figure 3:**
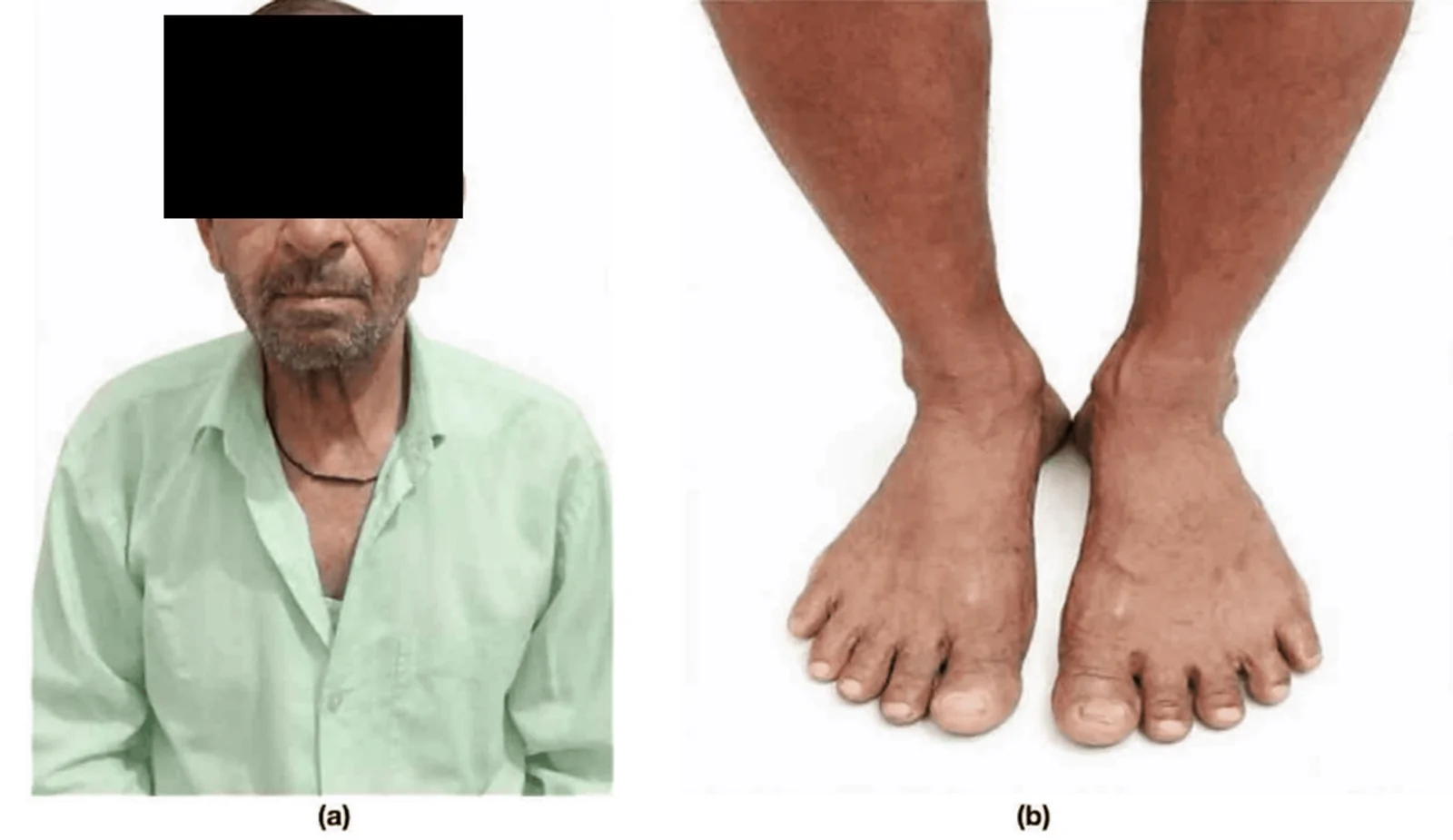
Complete clinical resolution after levothyroxine therapy a: face and upper trunk showing resolution of periorbital puffiness and facial swelling; b: both feet showing complete resolution of pedal edema. Photographs were taken at outpatient review approximately two months after initiation of levothyroxine. Written informed consent to include this image in an open-access article was obtained from the patient.

The overall diagnostic pathway followed in this patient, and the point at which thyroid function testing altered the trajectory of the work-up, are summarized in Figure 4.

**Figure 4:**
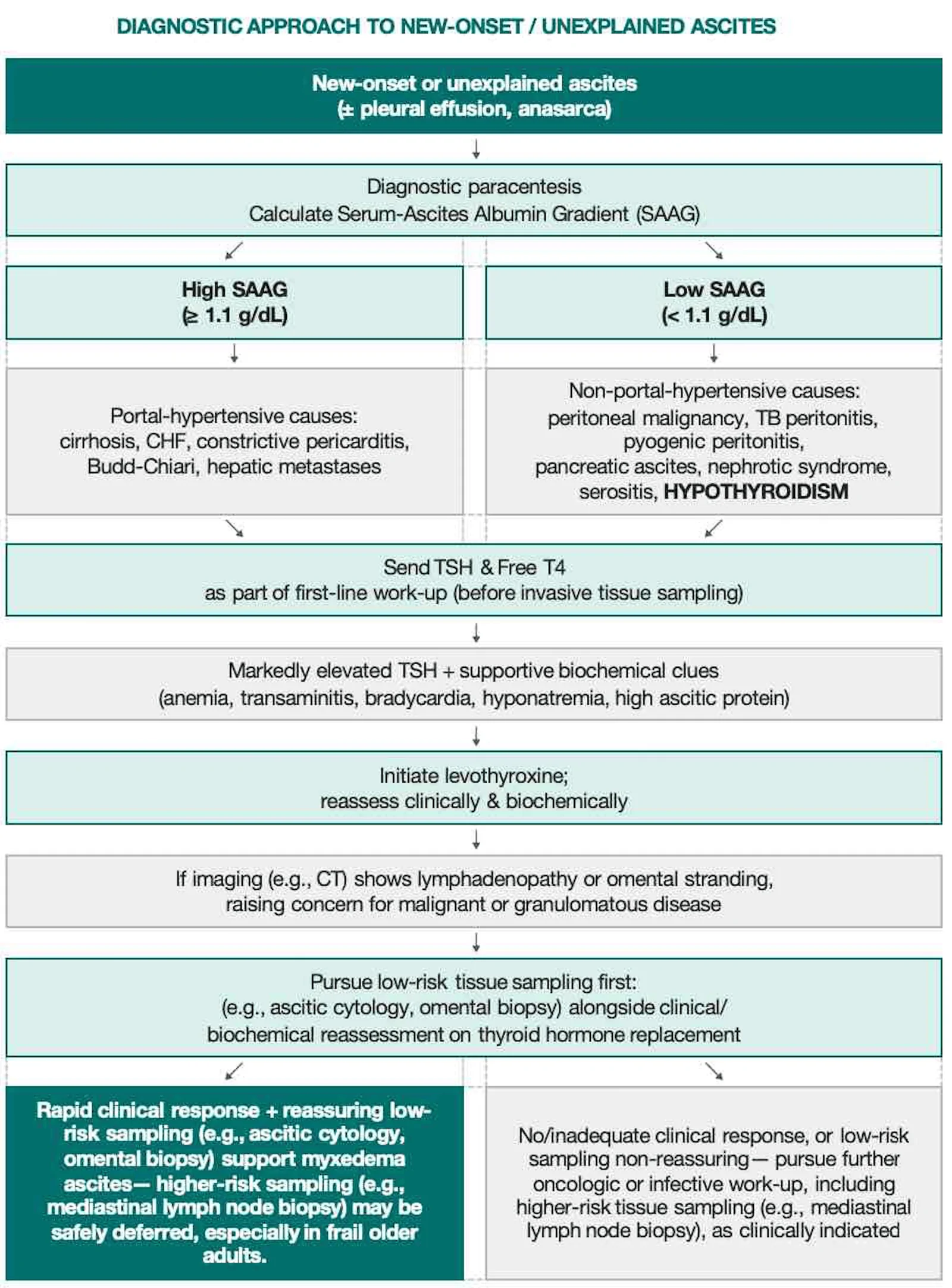
Diagnostic algorithm for ascites of undetermined etiology, incorporating thyroid function testing and risk-stratified tissue sampling SAAG: serum-ascites albumin gradient; CBNAAT: cartridge-based nucleic acid amplification test; TB: tuberculosis; TSH: thyroid-stimulating hormone

## Discussion

The pathophysiology of hypothyroidism-induced ascites remains incompletely understood. Proposed mechanisms include increased capillary permeability from low circulating thyroid hormone levels, leading to extravasation of plasma proteins into the extravascular and peritoneal compartments; a hygroscopic effect from hyaluronic acid-albumin complexes that impair lymphatic drainage of extravasated protein; diminished free-water clearance from relative excess of antidiuretic hormone; and, more recently, a role for reduced nitric oxide and elevated vascular endothelial growth factor levels driving endothelial dysfunction and further capillary leak [1,11]. Several of these mechanisms, protein extravasation, impaired free-water clearance, and endothelial dysfunction, are plausible contributors in this patient, whose course was notable not only for exudative, protein-rich ascites but also for progressive hyponatremia with inappropriately concentrated urine, a recognized biochemical accompaniment of severe hypothyroidism.

Ascitic fluid analysis is central to establishing the diagnosis and, as in this case, typically shows an elevated protein content (>2.5 g/dL), while the SAAG is variable and can be low or high depending on the relative contribution of capillary leak versus any coexistent portal hypertensive component. A high SAAG (≥1.1 g/dL) points toward a portal hypertensive cause with high diagnostic accuracy, whereas a low SAAG, as seen in this patient (0.5 g/dL), favors non-portal hypertensive causes and widens the differential to include peritoneal malignancy, tuberculous or pyogenic peritonitis, pancreatic ascites, nephrotic syndrome, and serositis [4,10,12], precisely the alternative diagnoses that this patient’s radiological findings of pleural effusion and mediastinal lymphadenopathy raised, and that required systematic re-evaluation, repeat ascitic fluid analysis together with cytology, tumor markers, and CBNAAT, before myxedema ascites could be accepted as the unifying diagnosis (Figure 4).

This case also illustrates several of the recognized biochemical clues to underlying hypothyroidism: normocytic anemia, transaminase elevation (attributable to thyroid-associated hepatic and muscle effects) [13], and bradycardia with low-voltage ECG complexes, all of which improved in parallel with biochemical response to thyroxine replacement. The presence of bilateral pleural effusion with mediastinal lymphadenopathy on CT, an atypical and, to our knowledge, less commonly emphasized radiological accompaniment of myxedema, appears to have contributed to diagnostic uncertainty in this frail older patient, underscoring how myxedema ascites can mimic malignant or granulomatous disease on imaging even when the underlying process is a reversible endocrinopathy. In older adults specifically, in whom the differential for new ascites is broad and comorbid frailty raises the stakes of invasive testing, thyroid function should be assessed as a routine part of the initial work-up, in parallel with, rather than after, the standard hepatic, cardiac, infective, and malignant evaluation. What distinguishes this case from most prior reports of myxedema ascites is the diagnostic uncertainty created by the radiological findings and the subsequent decision to defer further, higher-risk invasive tissue sampling (mediastinal lymph node biopsy). Once malignancy and tuberculosis had been reasonably de-prioritized through less invasive fluid investigations (cytology, tumor markers, and CBNAAT) and a lower-risk omental biopsy showing only non-specific inflammatory change without granuloma, necrosis, or atypia, and thyroid hormone replacement produced an early objective clinical response, further higher-risk mediastinal lymph node sampling was deferred by multidisciplinary consensus. In a frail older adult, in whom procedural risk and the burden of hospitalization may be substantial, a closely monitored therapeutic trial of thyroid hormone replacement may therefore be considered when the clinical trajectory is consistent with a reversible endocrine cause.

Consistent with the literature, this patient’s ascites and pleural effusion resolved completely with thyroid hormone replacement alone, without the need for diuretics, further paracentesis, or additional invasive intervention, reaffirming that myxedema ascites, however alarming its radiological mimicry of malignancy, is a reversible condition once euthyroidism is restored.

## Conclusions

Severe, previously undiagnosed hypothyroidism is a reversible cause of ascites that can present with pleural effusion and radiological lymphadenopathy mimicking malignancy or disseminated tuberculosis, particularly in older adults who are more likely to undergo an extensive alternative workup. This case reinforces the value of including thyroid-stimulating hormone testing as part of the initial evaluation of unexplained ascites and demonstrates that low-SAAG exudative ascites with markedly elevated protein content, in the setting of biochemical clues such as anemia, transaminitis, bradycardia, and hyponatremia, should prompt consideration of myxedema ascites before pursuing more invasive investigations. In this frail older adult, prompt initiation and titration of thyroid hormone replacement led to complete clinical resolution, and a closely monitored therapeutic trial, together with reassuring lower-risk tissue sampling (omental biopsy), allowed the higher-risk mediastinal lymph node biopsy to be safely avoided once the combined clinical, biochemical, and histological picture had reasonably lowered the likelihood of malignancy and infection, a strategy that merits wider consideration when comparable radiological mimicry of malignancy is encountered in frail patients with biochemical evidence of severe hypothyroidism.
